# In Vitro Anti-HIV-1 Activity of Chitosan Oligomers *N*-Conjugated with Asparagine and Glutamine

**DOI:** 10.3390/biotech12010018

**Published:** 2023-02-08

**Authors:** Fatih Karadeniz

**Affiliations:** Marine Biotechnology Center for Pharmaceuticals and Foods, College of Medical and Life Sciences, Silla University, Busan 46958, Republic of Korea; karadenizf@silla.ac.kr

**Keywords:** chitosan oligosaccharide, gp120, HIV-1, p24, phosphorylation

## Abstract

Chitosan oligomers (COS) are polysaccharides obtained by the hydrolyzation of chitosan. They are water-soluble, biodegradable, and have a wide range of beneficial properties for human health. Studies have shown that COS and its derivatives possess antitumor, antibacterial, antifungal, and antiviral activities. The goal of the current study was to investigate the anti-human immunodeficiency virus-1 (HIV-1) potential of amino acid-conjugated COS compared to COS itself. The HIV-1 inhibitory effects of asparagine-conjugated (COS-N) and glutamine-conjugated (COS-Q) COS were evaluated by their ability to protect C8166 CD4+ human T cell lines from HIV-1 infection and infection-mediated death. The results show that the presence of COS-N and COS-Q was able to prevent cells from HIV-1-induced lysis. Additionally, p24 viral protein production was observed to be suppressed in COS conjugate-treated cells compared to COS-treated and untreated groups. However, the protective effect of COS conjugates diminished by delayed treatment indicated an early stage inhibitory effect. COS-N and COS-Q did not show any inhibitory effect on the activities of HIV-1 reverse transcriptase and protease enzyme. The results suggest that COS-N and COS-Q possess an HIV-1 entry inhibition activity compared to COS and further studies to develop different peptide and amino acid conjugates containing N and Q amino acids might yield more effective compounds to battle HIV-1 infection.

## 1. Introduction

Natural polysaccharides are the most abundant natural polymers composed of sugar monomers linked to each other with glycosidic bonds. Up to date studies showed that polysaccharides possess health beneficial effects, not only in terms of nutrition but also as therapeutic agents. This led to the ever increasing attention focusing on polysaccharides to develop pharmaceuticals of novel natural origin. Starting with the first reported antiviral polysaccharides against mumps and influenza [1,2], several other reports have suggested that polysaccharides and their derivatives are potent antivirals [3]. The inhibition of human immunodeficiency virus 1 (HIV-1) by polysaccharides and their derivatives was also one of the reported antiviral activities [4,5,6]. Studies have also shown that sulfated polysaccharides from different sources showed beneficial effects against HIV infection either inhibiting cell entry or viral replication [7]. Additionally, reports of polysaccharide derivatization showed that inactive polysaccharides could present antiviral properties in vitro following the addition of different side chains to sugar monomers [8].

In this context, chitin is a polymer found abundantly in nature formed by *N*-acetylglucosamine units. It is arguably one of the most abundant polysaccharides after cellulose. It can be found in a broad range of organisms and in various tissues ranging from the shells of crustaceans to the fungi cell wall and even in some types of algae [9]. Chitin also constitutes a very big part of seafood processing by-products and has been reported to show some beneficial effects which cannot be utilized due to its insolubility in water. Chitosan is a derivative of chitin obtained by *N*-deacetylation. Chitosan is known to be a biologically active compound and was shown to exert antibacterial, antiviral, filtering, pesticide, and anti-aging activities. The main motive for studying and developing new applications for chitosan by either derivatization or chemical modification stemmed from the fact that it is easily obtainable, cheap, largely nontoxic, and mostly biodegradable [10]. Chitosan oligomers (COS) are composed of β-(1→4) *D*-glucosamine units and hydrolyzed derivatives of chitosan. COS were found to be easily absorbed by the human body compared to chitosan, which is one of their superior properties compared to chitosan and chitin, in addition to their free reactive amino groups, smaller size, and water solubility [11]. Expectedly, reports have also shown that COS were effective bioactive agents with numerous bioactivities [12]. Several novel compounds were developed by conjugating COS with other bioactive chemicals or adding potent sidechains to COS to improve their bioactivities [13,14,15]. Chitosan and COS were already reported to be potential candidates for peptide drug delivery where they were easily conjugated with bioactive peptides as encapsulation agents [16,17]. In addition, there are reports suggesting that the addition of amide-containing groups could improve the anti-HIV activity [18].

Chitosan derivatives were credited with possessing antiviral properties partly due to their ability to bind cell receptor molecules as a function of their protonated amino groups, different acetylation degrees, and the modification of polycationic moieties. Being open to modification at these positions allows chitosan and its oligosaccharides to show affinity towards different chemical compounds varying from fatty acids, other sugars, and peptides to phytochemicals such as flavonoids and tannins [19,20]. Additionally, the mucoadhesive properties of chitosan enable it remain at the host mucus surface and exhibit antiviral activities for longer periods. Chitosan itself in large molecular weights (50–1000 kDa) with a 10–30% degree of acetylation was reported to inhibit the proliferation of bacteriophages [21], tobacco mosaic virus [22], murine norovirus, and feline calicivirus [23]. Lowering its molecular weight was also shown to add broader antiviral properties where chitosan with a molecular weight below 10 kDa exerted antiviral properties against the different subtypes of influenza virus [24]. Chemical modifications of chitosan and chitosan oligomers were also reported to be promising means of developing antiviral chitosan-based agents. *N*-(2-hydroxypropyl)-3-trimethylammonium chitosan chloride inactivated human coronaviruses [25] while the sulfonation of both chitin [26] and chitosan oligomers [27] enhanced the antiviral activities against HIV-1. Nishumura et al. [26] suggested that sulfate moieties at the third and second O- of chitin increased their affinity towards negatively charged gp120 to disrupt virus–host interactions. Although conjugating chitosan with peptides and proteins has mostly been regarded as a drug delivery or non-viral gene transfection approach, studies have shown that chitosan derivatization that adds short peptide chains or amino acids is beneficial in terms of enhancing bioactivity. Lv et al. [28] showed that *N*-arginine-chitosan was able to enhance the antiviral efficiency of drug adefovir. Jaber et al. [29] also reviewed the antiviral potential of chitosan–arginine derivatives and reported a broad antiviral activity against H1N1 influenza A, the Copenhagen strain of vaccinia virus, and SP7 HPS-1.

In the present study, COS were *N*-conjugated with amino acids with amide-containing side chains: asparagine and glutamine. The in vitro anti-HIV-1 activity of asparagine- and glutamine-conjugated COS were investigated in human T cells infected with different strains of HIV-1 and their anti-HIV activity was compared to COS.

## 2. Results and Discussion

### 2.1. Inhibition of HIV-1 Infection

#### 2.1.1. Inhibition of Syncytia Formation and HIV-Induced Lytic Effects

**Figure 1:**
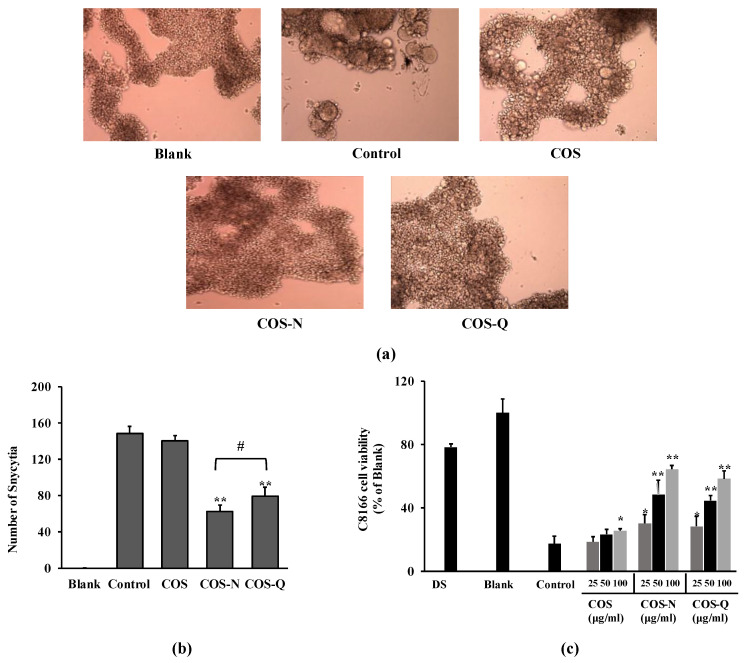
Effect of COS conjugates on HIV-1 infection of C8166 cells. (**a**) Images of C8166 cells infected with or without HIV-1_RF_ at day 2 post-infection. Infected cells were treated with COS or COS conjugates at the concentration of 100 μg/mL. Blank: Uninfected untreated cells, Control: HIV-1-infected untreated cells. (**b**) Number of syncytia counted at day 2 post-infection via optical microscope. (**c**) Effect of COS conjugates on HIV-1-induced lytic effect measured by cell viability. Viability of cells given as a relative percentage of Blank (uninfected untreated) group measured at day 5 post-infection. DS: dextran sulfate, 100 μg/mL. * *p* < 0.05, ** *p* < 0.01 vs. Control (HIV-1-infected untreated). ^#^ *p* < 0.05 COS-N vs. COS-Q.

Primarily, asparagine-conjugated COS (COS-N) and glutamine-conjugated COS (COS-Q) were evaluated for their anti-HIV-1 properties by their potential to inhibit syncytia formation on C8166 human T cells infected with syncytia inducing X4 tropic HIV-1 strain (HIV-1_RF_). Microscopic images of HIV-1_RF_-induced syncytia inhibitory activity of compounds are shown in Figure 1a. Syncytia formation on C8166 cells was observed and recorded at day 2 post-infection. Cells infected with HIV-1 produce a high amount of membrane proteins specific to HIV-1 binding which then interact with other infected cells and fuse with each other, resulting in a giant cell formation called syncytia [30]. According to the images of cells and the quantification of formed syncytia, COS-N and COS-Q were both able to reduce the HIV-1-induced syncytia formation. Counting syncytia formations revealed that the COS-N-treated cells exhibited a smaller syncytia count than those treated with COS-Q (Figure 1b). Both COS-Q- and COS-N-treated cells exhibited less syncytia formation compared to COS-treated cells.

The conjugated COS were examined for their ability to protect C8166 cells from HIV-1-induced lysis. The formation of syncytia is followed by cell killing due to the bursting of multinucleated giant cells. The effects were analyzed by cell viability five days after infection. The results show that untreated cells showed 17.24% viability at day 5 post-infection compared to the untreated uninfected group. Treatment with COS-N and COS-Q dose-dependently decreased the HIV-induced cell lysis shown as increased viability (Figure 1c). At the concentration of 100 μg/mL, the COS-N-treated group showed 64.24% viability. This number was 58.21% for the COS-Q-treated cells. Dextran sulfate (DS) was used as a positive control (100 μg/mL) and showed a similar effect to COS-N, although higher with 78.24% viability. On the other hand, COS-treated cells showed 25.43% viability.

#### 2.1.2. Inhibition of the HIV-1 p24 Antigen

**Figure 2:**
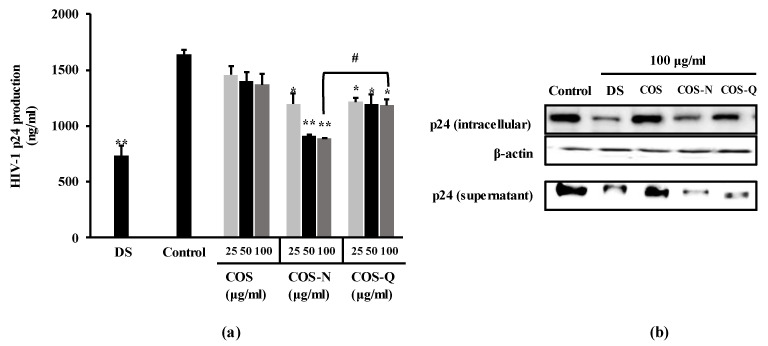
Effect of COS conjugates on the HIV-1 p24 production in HIV-1_IIIB_-infected H9 cells. (**a**) p24 release in HIV-1-infected H9 cell culture medium was measured by p24 capture ELISA and given as ng per ml of supernatant. (**b**) Intracellular and supernatant p24 levels were detected by Western blot. DS: dextran sulfate, 100 μg/mL; Control: HIV-1-infected untreated cells. * *p* < 0.05, ** *p* < 0.01 vs. Control (HIV-1-infected untreated). ^#^ *p* < 0.05 COS-N vs. COS-Q. (Original figures: Figure S1)

In order to further confirm the anti-HIV-1 effect of the COS-N and COS-Q, the production of the p24 antigen, one of the main viral markers of HIV-1 infection, was measured by ELISA and Western blot. The p24 protein is a crucial viral marker usually tested to diagnose HIV-1 infection along with HIV-1 RNA and antibodies. It is the capsid protein that makes up most of the protein load of the HIV-1 virus. In this experiment, H9 cells were used due to this cell line being permissive for HIV-1 replication for longer periods compared to the other cell lines used in this assay. As the aim of current investigation was to detect p24 antigens, H9 cells were infected with the HIV-1_IIIB_ strain instead of syncytia-inducing HIV-1_RF_ and C8166 cells. The results of p24 antigen capture ELISA show that HIV-1-infected H9 cells expressed a significant amount of p24 antigen (Figure 2a) which was suppressed by both COS-N and COS-Q treatment. At the concentration of 100 μg/mL, the supernatants obtained from COS-N-treated cell culture medium contained 890.43 ng/mL p24 antigen compared to 1640.36 ng/mL of untreated infected cells. The COS-Q-treated cell culture supernatant contained 1183.49 ng/mL while DS (100 μg/mL) treatment resulted in 732.07 ng/mL p24 release. COS-treated only cells were measured to release 1368.90 ng/mL p24.

This was in parallel to the Western blot results where the cellular p24 levels and p24 levels of supernatant were dose-dependently decreased by COS-N and COS-Q treatment (Figure 2b). This suggested that the treatment with COS conjugates decreased the viral production of HIV-1 proteins.

### 2.2. Inhibition of HIV-1 Reverse Transcriptase (RT) and Protease Activity

To provide insights into the action mechanism of the COS-N and COS-Q anti-HIV effect, their ability to inhibit the enzymatic activities of HIV-1 RT and protease was investigated. Previous results indicating that COS conjugates were able to decrease HIV-1 reproduction, however, are not indicative of whether COS conjugates intervened in the step of the HIV-1 viral cycle. Some of the main drugs on the market such as azidothymidine and saquinavir are enzyme inhibitors. These inhibit HIV-1 RT to stop viral gene insertion or HIV-1 protease to stop HIV-1 protein maturation that forms new infectious virions [31,32]. Several natural products, including polysaccharides and their derivatives, were reported to inhibit one of the enzymes to exert their anti-HIV-1 activity [7,33,34]. Therefore, to elucidate the action mechanism of COS conjugate-mediated HIV-1 inhibition, initially, HIV-1 RT and protease inhibitory effects of COS-N and COS-Q were evaluated.

**Figure 3:**
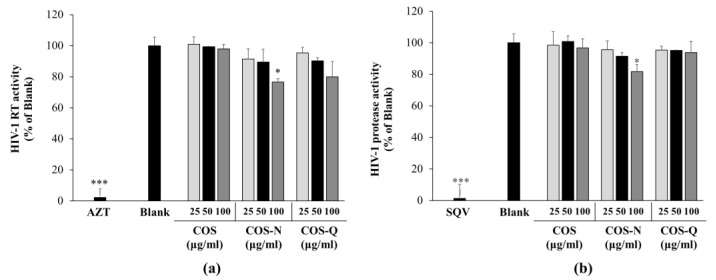
Effect of COS conjugates on the HIV-1 RT (**a**) and HIV-1 protease (**b**) activity. Enzymatic activities of HIV-1 RT and protease were measured with commercial assay kits as directed. Azidothymidine (AZT, 5 μM) and saquinavir (SQV, 5 μM) were used, and RT and protease inhibitors were the positive controls, respectively. Blank: HIV-1 RT or protease enzyme without any treatment. * *p* < 0.05 and *** *p* < 0.001 vs. Blank (only enzyme, no treatment).

The results show that although COS-N and COS-Q both inhibited the HIV-1 RT to some extent at 100 μg/mL (23.32% for COS-N and 20.14% for COS-Q), it was not comparable to that of the azidothymidine (5 μM) treatment which almost completely inhibited the HIV-1 RT (97.81%) (Figure 3a). COS treatment did not show any effect on HIV-1 RT activity. Similar results were obtained with the protease activity assay (Figure 3b). The COS-Q and COS treatments were not inhibitory to HIV-1 protease. COS-N treatment inhibited 18.30% of the protease activity of the untreated control, whereas treatment with saquinavir (5 μM) almost completely inhibited the HIV-1 protease (98.61%). These results suggest that COS conjugates are not inhibitors of HIV-1 enzymes. Studies have shown that some other anti-HIV-1 compounds with amide derivatization acted as entry inhibitors of human viruses including HIV-1 blocking the virus–cell or cell–cell connections to inhibit the entry of the virus into the host cell [35,36,37]. Some of these compounds were only able to inhibit HIV-infection at the early stages and did not show any effect in later stages. Expectedly they did not show any inhibitory effect on HIV-1 RT, protease, and integrase. According to the obtained data, and the reports of entry inhibitors with amide derivatization, it was hypothesized that COS conjugates might be effective at the early stages of HIV-1 infection.

### 2.3. Inhibition of HIV-1 Entry

**Figure 4:**
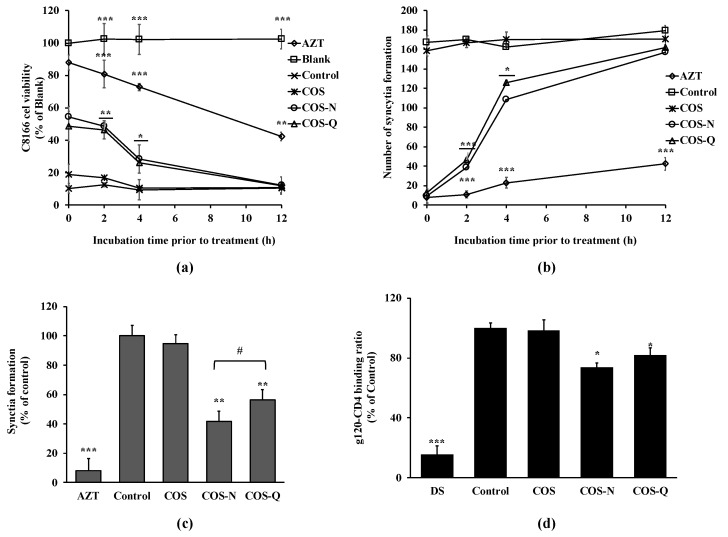
Effect of delayed addition of COS conjugates on HIV-1-induced lytic effects and syncytia formation. (**a**) C8166 cells were infected with HIV-1_RF_ and incubated for 0, 2, 4, and 12 h before being treated with COS conjugates or AZT (azidothymidine, 5 μM). Viability of cells was measured by MTT assay at day 5 post-infection. (**b**) HIV-1_RF_-infected C8166 cells were incubated for 0, 2, 4, and 12 h before being treated with COS conjugates or AZT (azidothymidine, 5 μM). Syncytia formations were counted at day 2 post-infection. (**c**) Uninfected C8166 cells were co-cultured with H9 cells infected with HIV-1_IIIB_ at a ratio of 10:1 and treated with COS conjugates (100 μg/mL) or AZT (azidothymidine, 5 μM). Syncytia formation was counted at day 5 post-infection. Blank: Uninfected untreated cells, Control: HIV-1-infected untreated cells. * *p* < 0.05, ** *p* < 0.01, and *** *p* < 0.001 vs. Control. (**d**) Effect of COS and COS conjugates (100 μg/mL) on HIV-1 gp120 binding with host cell receptor CD4 was investigated by ELISA. Binding was given as a relative percentage of the Control group where gp120 and CD4 interaction did not interfere with any treatment. * *p* < 0.05, ** *p* < 0.01, and *** *p* < 0.001 vs. Control; ^#^ *p* < 0.05 COS-N vs. COS-Q.

To further understand the anti-HIV-1 mechanism of COS conjugates, a delayed addition experiment was carried out. The delayed addition of COS conjugates to the infected cells was expected to provide insights into which stages of HIV-1 infection they might intervene in. The results show that adding COS conjugates to the cell culture resulted in a time-dependent decrease in cell viability (Figure 4a). Starting treatment with COS-N and COS-Q at 12 h post-infection did not exert any protective effect against HIV-1-induced lysis. These results are in parallel with those of syncytia formation (Figure 4b). At two days post-infection, both COS conjugate-treated and untreated cells exhibited the same amount of syncytia formation. These results indicate that COS-N and COS-Q affected HIV infection at the early stages and probably intervened in host entry stage.

The co-culture experiment was also performed to further understand the HIV-1 entry inhibitory effect of COS-N and COS-Q. C8166 cells were co-cultured with HIV-1IIIB chronically infected H9 cells. The cell-to-cell interaction between the gp120 protein of infected cells and CD4 receptors of infected cells were expected to mimic HIV-1 infection in vivo and result in multinucleated giant cells and result in cell killing [30]. As seen in Figure 4c, syncytia formation was inhibited by 58.24% after COS-N treatment (100 μg/mL) and by 43.61% after COS-Q treatment (100 μg/mL). COS treatment was deemed ineffective to protect cell–cell fusion. This result shows that COS-N and COS-Q treatment was able to inhibit cell–cell fusion. The entry stage of the HIV-1 viral cycle starts with the binding of HIV-1 gp120 protein to a specific receptor on T cells called CD4 and its co-receptors. Therefore, the effect of COS conjugates on the cell–cell fusion was further investigated with ELISA. As seen in Figure 4d, the COS-N and COS-Q treatment significantly hindered the binding of gp120 with CD4. DS was used as a positive control due to its reported HIV-1 entry inhibitory properties [27]. Although the inhibitory effect of COS conjugates was lower than that of DS (81.61%), the results indicate that the anti-HIV-1 effect of COS-N and COS-Q might exhibit itself by disrupting the interaction between the virus and host cell. Yoshida [38] suggests that the sulfate groups in sulfated polysaccharides might interact with positively charged amino acids in HIV-1 gp120. Similarly, Battulga et al. [4] reported the interaction between sulfated polysaccharides and HIV-1 surface protein, gp120. Although N- and Q-conjugated COS did not possess the negative charges of sulfation, Jeon and Kim [39] postulated that positive charges of COS amino acid conjugates might interact with negative charges on the cell surface. In this case, COS-N and COS-Q might compete with gp120 to bind the cell surface receptor-sulfated polysaccharides to inhibit viral entry as Crublet et al. [40] reported that gp120 of HIV-1 showed affinity to cell-associated sulfated groups, which consequently increased the viral infectivity. On the other hand, modification with polar amino acids is expected to increase the solubility of COS, and therefore, enhance the interaction between viral proteins and compounds. This was consistent with the present results which suggest that COS conjugated with amino acids inhibited HIV-1 infection by interfering with virus–host cell binding.

## 3. Materials and Methods

### 3.1. Materials

Chitosan oligomers (<1 kDa) were obtained from Kitto Life (Seoul, Korea). For the syntheses of COS derivatives *N*-conjugated with asparagine (N) and glutamine (Q), their *N*-t-tert-butyloxycarbonyl (Boc) amino acid derivatives were purchased from Sigma Chemical Co. (St. Louis, MO, USA). N,N′-Dicyclohexylcarbodiimide (DCC), triethylamine (TEA), and trifluoroacetic acid (TFA) were also from Sigma Chemical Co. (St. Louis, MO, USA). Remaining reagents and chemicals were purchased from Junsei Chemical (Tokyo, Japan). The reagent 3-(4,5-dimethylthiazol-2-yl)-2,5-diphenyltetrazolium bromide (MTT) and dimethyl sulfoxide (DMSO) were bought from Sigma Chemical Co. (St. Louis, MO, USA). Necessary items for cell culture and maintenance (medium-RPMI 1640, penicillin/streptomycin, fetal bovine serum, etc.) were purchased from Gibco BRL (Grand Island, NY, USA). H9 cell line was from the American Type of Culture Collection (Manassas, VA, USA). C8166 cell line and HIV-1_RF_ strain were provided by Dr. G. Farrar from the EU Programme EVA Centre for AIDS Reagents, NIBSC, UK.

### 3.2. Addition of Amino Acids to COS

*N*-conjugation of asparagine and glutamine was carried out as previously reported [39]. Briefly, conjugates were prepared by the *N*-conjugation of Boc bound amino acids (Boc-AA) to the C-2 position of the chitosan oligomer (COS) monomers. Then, 25 g of COS with approx. 1 mM free amino group was dissolved in 50 mL water. Two hundred milliliters of methanol was added to COS and the pH of the mixture was adjusted to pH 6.8 with TEA. Mixture was put into shaker for 24 h after the addition of 100 mM Boc-AA and 100 mM dicyclohexyl carbodiimide (DCC) as the coupling agent. After 24 h, the mixture was filtered and kept overnight at 2 °C. Boc-AA-COS was removed from the mixture by precipitation via the addition of an adequate amount of ether, before being filtered and lyophilized. Finally, Boc protection was removed to obtain COS-AA. Asparagine-conjugated COS (COS-N) and glutamine-conjugated COS (COS-Q) were analyzed for the degree of substitution for COS-N: 0.78; and for COS-Q: 0.53, which were calculated from elemental analysis as described.

### 3.3. Cell Culture, HIV-1 Infection, Syncytia Formation, and Cell Viability Analysis

H9, H9/HIV-1_IIIB_, and C8166 cell lines were propagated at 37 ℃ under 5% CO_2_ in complete RPMI 1640 medium supplemented with 10% FBS, 100 μg of streptomycin per ml, and 100 U of penicillin per ml. All cells were cultured in either T25 or T75 cell culture flasks. Cells were sub-cultured 2–3 times a week (1 × 10^5^ cells/T-25 flask). Cells were discarded every 2 months and replaced from fresh stocks. Prior to experiments, cells were transferred into 48-well plates unless otherwise noted.

HIV-1 infection of human T cell line C8166 cell was followed by large, multinucleated giant cell formations called syncytia. These syncytia formations eventually led to cell death after swelling. Basically, C8166 cells were seeded in 48-well plates (1 × 10^5^ cells/well) in the presence or absence of different concentrations of COS conjugates in RPMI-1640 with 5% FBS. Cells were infected after 2 h incubation with HIV-1_RF_ (100 μL) diluted in RPMI-1640 to 200 CCID_50_ (μg/mL). Cells were kept at 37 °C for 48 h and syncytia was confirmed and counted optically using an inverted microscope (DMire2, Leica Microsystems, Solms, Germany). For each treatment group, three wells were counted thrice and the average ± SD was noted as the final syncytia count.

To determine the protective effect of amino acid-conjugated COS against HIV-1 induced lysis in C8166 cells, an MTT-formazan-based cell viability assay was performed. Cells in log-growth phase (four days post-seeding) were harvested, washed, and added in 48-well plate (1 × 10^5^ cells/well) in the presence or absence of different concentrations of COS conjugates. Stock supernatants of HIV-1_IIIB_ were diluted RPMI-1640 to yield sufficient cytopathicity (~90% cell kill in 5 days) (CCID_50_) and used to infect the cells (100 μL per well). Plates were kept for 5 days at 37 °C and 100 μL MTT solution (500 μg/mL in PBS) was added to each well and cells were kept for a further 4 h at 37 °C. The formazan salt formed by viable cells was dissolved in acidified propanol containing 50% DMSO and 4% triton X-100. The absorbance values of each well were measured at 540 nm with a GENios microplate reader (Tecan, Austria). The absorbance value of untreated and uninfected cells was taken as 100% comparison viability and rest of the treatment groups were given as relative percentage.

### 3.4. Measurement of p24 Antigen

To measure the p24 antigen levels in HIV-1-infected cell culture medium, a commercial p24 antigen capture ELISA was used (Perkin-Elmer Life Sciences, Boston, MA, USA) following the manufacturer’s directions. Basically, H9 cells were cultured in 24-well plates at a density of 5 × 10^5^ cells/well and infected with HIV-1_RF_ as described in Section 3.3. Cells were treated with COS-conjugates for five days and the supernatants of the culture wells were used to detect the virus released to medium by p24 ELISA.

Expression of p24 protein was investigated by Western blotting in cellular fractions and culture medium. H9 cells were cultured and infected as described above. Cells were treated with COS-conjugates for five days. To analyze the protein levels of p24, the total protein was isolated from cells through the addition of 1 mL lysis buffer containing 50 mM Tris-HCl, 0.4% (*w*/*v*) NP-40, 120 mM NaCl, 1.5 mM MgCl_2_, 2 mM PMSF, 3 mM NaF, and 1 mM DTT to each well after harvesting the supernatant. Viral proteins were obtained from harvested supernatants as described earlier [27]. On the other hand, cell lysates were centrifuged at 12,000 rpm for 10 min and the supernatants were used for Western blotting. The protein concentration of the samples was calculated using the BCA protein assay kit (Thermo Fisher Scientific, Waltham, MA, USA) and twenty micrograms of protein from each well was loaded on a 10% SDS-PAGE gel. Following SDS-PAGE, the proteins were transferred to nitrocellulose membranes. Blotted membranes were then blocked in 5% skim milk for 4 h at room temperature and hybridized with p24 monoclonal primary antibody overnight at 4 °C. Membranes were then subjected to horse radish peroxidase conjugated anti-mouse antibody for 1 h at room temperature. Protein bands on membranes were visualized with a commercial chemiluminescence kit (Amersham ECL detection kit, GE Healthcare, Chicago, IL, USA) and the images were taken on a Fujifilm Imaging System (Fujifilm Life Science, Tokyo, Japan).

### 3.5. HIV-1 RT and Protease Activity Assay

The activity of HIV-1 reverse transcriptase, isolated from the virus pellet of H9/HIV-1_IIIB_ culture supernatant as described in earlier studies [27], was evaluated using a fluorescence RT assay kit (EnzChek, InvitroGen, Carslbad, CA, USA) according to the manufacturer’s protocol. The activity of the HIV-RT was measured as the fluorescence intensity of the wells as a result of RT activity on the substrate. Fluorescence intensity was measured at 480 nm (excitation) and 520 nm (emission) with a GENios microplate reader (Tecan Austria GmbH, Grodig, Austria) after the addition of 173 μL of fluorescent PicoGreen reagent prepared in TE buffer.

In order to assess the protease inhibitory effect of COS conjugates, a commercial SensoLyte 520 HIV-1 protease assay kit (Anaspec, CA, USA) was used according to manufacturer’s directions. COS conjugates were tested for their ability to inhibit the proteolytic cleavage of HiLyte Fluor™488/QXL™520 FRET peptide (obtained from the SensoLyte 520 HIV-1 protease assay kit) by HIV-1 protease. The amount of HiLyte Fluor™488 produced by successful protease activity was measured by GENios^®^ microplate reader (Tecan Austria GmbH, Austria).

### 3.6. Delayed Addition and Co-Culture Assays

C8166 cells were seeded in a 48-well plate (3 × 10^4^ cells/well). Cells were infected as previously described in Section 3.3 (syncytia formation count). After the addition of the virus, 100 μL of 100 μg/mL COS conjugates, saquinavir, and azidothymidine were added into multiple wells. Blank group was only treated with samples and incubated without being infected with the virus. After a total of 96 h incubation, cellular viability was assessed using the MTT assay, as previously described in Section 3.3, and the protection ability is calculated in reference to treated yet uninfected cell groups.

For the co-culture assays, C8166 and H9 cells were cultured together. Briefly, C8166 cells were seeded in a 48-well plate (5 × 10^4^ cell/well) along with H9 cells (infected with HIV-1_IIIB_ as described in Section 3.3) in the ratio of 10:1 in the presence or absence of COS conjugates. The cells were incubated for 48 h, and the number of syncytia was counted using a light microscope as noted in Section 3.3.

### 3.7. Analysis of gp120-CD4 Binding

The effect of COS conjugates on HIV-1 entry was investigated via its interaction with CD4-gp120 binding. ELISA was carried out as previously reported with the required modifications [30]. Briefly, the wells of an ELISA plate were coated with 2 μg/mL anti-gp120 antibody (Santa Cruz Biotechnology, Santa Cruz, CA, USA) in carbonate buffer (pH 9.6) and incubated overnight at 4 °C. Wells were then blocked with PBS containing 0.1% BSA at 37 °C for 1 h. Recombinant HIV-1_IIIB_ gp120 protein (100 ng/well) in PBS was added to each well and the plate was incubated at 37 °C for 1 h. The wells were then washed with PBS containing 0.05% Tween 20. Different concentrations of PCOS were added to each well along with human sCD4 (100 ng/well) and the plates were further incubated at 37 °C for 1 h. After the incubation wells were treated with anti-sCD4 IgG (250 ng/mL), another incubation followed at 37 °C for 1 h. Subsequently, the horseradish peroxidase conjugated with streptavidin was added for the detection of gp120-bound CD4 proteins. Measurement was carried out by detecting the absorbance values of wells at 405 nm after adding a water-soluble horseradish peroxidase substrate that produces the yellow color (*O*-phenylenediamine dihydrochloride).

### 3.8. Statistical Analysis

The statistical significance of experimental data was determined and expressed as a mean of three independent experiments ± standard deviation (SD). Differences between the means were analyzed using the analysis of variance (ANOVA) procedure of Statistical Analysis System, SAS v9.2 (SAS Institute, Cary, NC, USA) with Duncan’s multiple range test as a post hoc analysis. The significance of differences was defined at the *p* < 0.05 level.

## 4. Conclusions

In conclusion, the current results point out that COS-N and COS-Q possibly inhibited HIV-1 infection by blocking the connection between HIV-1 (or infected cells) and uninfected cells. However, DS and the other positive controls showed higher activity than COS conjugates, but the efficiency of COS conjugates did not generate further interest in developing them into antiviral agents. Nevertheless, the experiments showed that the *N*-conjugation of COS with amino acids enhances its antiviral capabilities and the amide group side chains added COS anti-HIV-1 properties. Further studies with longer peptide sequence conjugations and experiments that aim to elucidate the interaction between COS conjugates and the HIV-1 cell entry mechanism should yield valuable data towards the utilization of COS–protein conjugates as lead molecules for HIV-1 drug development.

## Data Availability

Data used to support the findings of this study are available from the corresponding author upon reasonable request.

## Supplementary Materials

The following are available online at https://www.mdpi.com/article/10.3390/biotech12010018/s1, Figure S1: Original Western blot images.
