# Supplementary figures and images for: In Vitro Anti-HIV-1 Activity of Chitosan Oligomers N-Conjugated with Asparagine and Glutamine
Source: BioTech (Basel). 2023 Feb 8;12(1):18. doi: 10.3390/biotech12010018 (PMC9944945; doi:10.3390/biotech12010018)

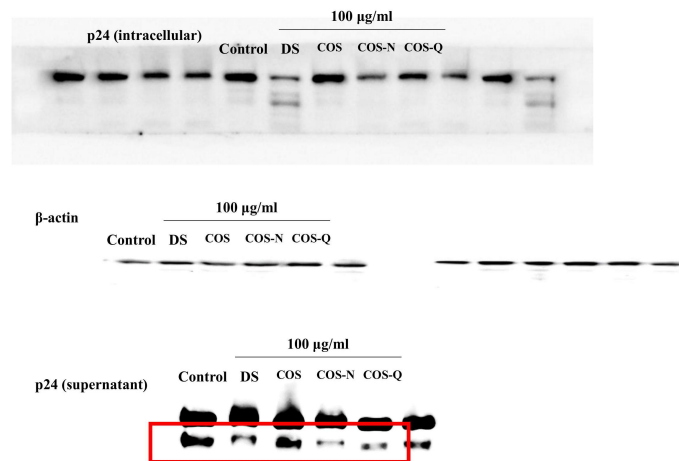

**Figure S1.** Original Western blot images.

Supplement: Supplementary file 1 [file biotech-12-00018-s001.zip › biotech-2165098-supplementary.pdf]
